# Health outcomes of patients with type 2 diabetes following bariatric surgery: Results from a publicly funded initiative

**DOI:** 10.1371/journal.pone.0279923

**Published:** 2023-02-24

**Authors:** Trisha O’Moore-Sullivan, Jody Paxton, Megan Cross, Srinivas Teppala, Viral Chikani, George Hopkins, Katie Wykes, Paul A. Scuffham, on behalf of the Clinical and Operational Reference Group

**Affiliations:** 1 Medical and Chronic Disease Services, Mater Health, Brisbane, Queensland, Australia; 2 Healthcare Improvement Unit, Clinical Excellence Queensland, Queensland Health, Brisbane, Queensland, Australia; 3 Menzies Health Institute Queensland, Griffith University, Gold Coast, Queensland, Australia; 4 Department of Diabetes and Endocrinology, The Princess Alexandra Hospital, Brisbane & Senior Lecturer, University of Queensland, Brisbane, Queensland, Australia; 5 Royal Brisbane and Womens Hospital, Queensland Health, Brisbane, Queensland, Australia; University of Toronto, CANADA

## Abstract

**Objective:**

Bariatric surgery is an effective treatment for type 2 diabetes and morbid obesity. This paper analyses the clinical and patient-reported outcomes of patients treated through the Bariatric Surgery Initiative, a health system collaboration providing bariatric surgery as a state-wide public service in Queensland, Australia.

**Research design and methods:**

A longitudinal prospective cohort study was undertaken. Eligible patients had type 2 diabetes and morbid obesity (BMI ≥ 35 kg/m^2^). Following referral by specialist outpatient clinics, 212 patients underwent Roux-en-Y gastric bypass or sleeve gastrectomy. Outcomes were tracked for a follow-up of 12-months and included body weight, BMI, HbA1c, comorbidities, health-related quality of life, eating behaviour, and patient satisfaction.

**Results:**

Following surgery, patients’ average body weight decreased by 23.6%. Average HbA1c improved by 24.4% and 48.8% of patients were able to discontinue diabetes-related treatment. The incidence of hypertension, non-alcoholic steatohepatitis, and renal impairment decreased by 37.1%, 66.4%, and 62.3%, respectively. Patients’ emotional eating scores, uncontrolled eating and cognitive restraint improved by 32.5%, 20.7%, and 6.9%, respectively. Quality of life increased by 18.8% and patients’ overall satisfaction with the treatment remained above 97.5% throughout the recovery period.

**Conclusions:**

This study confirmed previous work demonstrating the efficacy of publicly funded bariatric surgery in treating obesity, type 2 diabetes and related comorbidities, and improving patients’ quality of life and eating behaviour. Despite the short follow-up period, the results bode well for future weight maintenance in this cohort.

## Introduction

The link between type 2 diabetes and obesity is well established [1–3], as is the potential remediation of diabetes through weight loss supported by metabolic surgery [4–6]. In Australia, the prevalence of type 2 diabetes is increasing; an estimated 4.1% of the population (998,100 people) had type 2 diabetes in 2017–18 (accounting for 11% of 2018 deaths), compared to 3.3% in 2001 [7]. Obesity rates have also increased, tripling from 8% to 24% in the past 30 years [8]. The resulting impact on health expenditure has been substantial; compared with those of normal weight, costs are 26% higher for individuals with obesity, 46% higher for those with both obesity and diabetes, and may be up to 51% higher if additional obesity-related comorbidities are present [9, 10].

In Queensland, Australia, 32% of adults self-reported as having obesity in 2020 and 54% of the health burden from type 2 diabetes was attributed to overweight and obesity (66% combined) [11]. Bariatric surgery is the most effective treatment for morbid obesity [12]. Several long-term global surveys confirm the effectiveness of bariatric surgery on weight loss, reduction of obesity-related complications, increased quality of life [13–17], and treatment of type 2 diabetes [18]. The consequent popularity of bariatric surgery has generated high demand [19], but provision of a publicly funded bariatric surgery program requires careful resource allocation [20] and Australian states and territories’ prioritisation processes for the procedure are limited and highly inconsistent [21].

In response to the need to use the available and finite resources to achieve maximum benefit for patients, structures and processes were established to deliver bariatric surgery in the public system to patients with type 2 diabetes and morbid obesity who are seeking assessment for surgical intervention. The Queensland Department of Health funded the Bariatric Surgery Initiative (BSI), a collaboration between Metro South Hospital and Health Service (MSHHS), Metro North Hospital and Health Service (MNHHS), Clinical Excellence Queensland (CEQ) and the Healthcare Purchasing and System Performance Division (HPSP) of Queensland Health. Between 2017 and 2020, the BSI developed and evaluated a central referral process, a standardised referral form with inclusion/exclusion criteria, and an evidence-based clinical assessment and prioritisation instrument, the Bariatric Surgery Assessment and Prioritisation Tool (BAPT, see S1 File), which will be the subject of a forthcoming manuscript. Patients referred to the program were assessed using the BAPT and were prioritised for surgery based on their clinical need and likelihood of benefitting from the procedure. Following surgery, patients’ clinical outcomes and self-reported experiences were recorded for 12 months to examine changes in their health status and quality of life. The aim of this article is to report the baseline and initial outcomes of weight loss, diabetes, obesity-related comorbidities, and quality of life, along with their satisfaction with the service and self-reported changes to eating behaviour.

## Research design and methods

### Study design

A longitudinal prospective cohort study design was used in accordance with STROBE guidelines [22]. The clinical dataset used for this evaluation was collected on 30 September 2020; it includes patient data collected between the first referral in September 2017 and the most recent follow-up in September 2020. It is important to note that the program was interrupted due to the COVID-19 pandemic. Due to a finite funding window, data collection could not be extended beyond the initial study timeframe and thus, some patients had not yet reached the 12-month follow up point when the study concluded. Data were de-identified prior to analysis and stored in accordance with the Australian Code for Responsible Conduct of Research.

#### Ethics

Ethics approvals for the evaluation were received from the Metro South Hospital and Health Service and Griffith University Human Research Ethics Committees on 20 September 2018 and 2 August 2019, respectively, with additional amendments approved on 18 January 2021 (Project HREC/18/QPAH/427). Written informed consent was obtained from all patients for their participation in the study, for release of their de-identified clinical data to Griffith University for the study and for them to undergo bariatric surgery. Additional data were obtained from Queensland Health following application under the *Public Health Act 2005*.

#### Patient recruitment and surgical procedures

Based on clinical evidence, inclusion and exclusion criteria were established by an expert Clinical and Operational Reference Group (CORG) to screen patients prior to assessment (see Supplementary material). Briefly, inclusion in the BSI cohort required patients to: have a body mass index (BMI) ≥ 35 kg/m^2^; have type 2 diabetes with glycated haemoglobin (HbA1c) > 7%, despite treatment with oral medications and/or insulin; be aged 18–65 years; and be under the care of a public hospital specialist for conditions that may be improved by bariatric surgery.

Patients were referred to the BSI by specialist outpatient clinics from 25 September 2017 to 9 August 2019. Following multidisciplinary assessment of each patient, the BSI team determined whether patients continued to surgery or were referred back to a specialist for other forms of medical management. Of the 297 referrals received, 212 patients proceeded to surgery in this evaluation period and underwent either Roux-en-Y gastric bypass (RYGB) or sleeve gastrectomy (SG) at the Queen Elizabeth II Jubilee Hospital (MSHHS) or Royal Brisbane and Women’s Hospital (MNHHS).

#### Post-surgery assessment and data collection

The post-surgical model of care comprised post-operative telephone follow-ups at 2 weeks with a nurse and dietitian, and 1-, 3-, 6- and 12-month post-op reviews with the surgeon, dietitian, endocrinologist, and psychologist. Patient outcomes were collected at these time points by service coordinators at the sites and submitted to a centralised data repository managed by Queensland Health.

*Patient outcomes data*. Body weight, BMI, HbA1c, diabetes medications, and other chronic disease factors, including changes in lipid status (dyslipidaemia), liver function (non-alcoholic steatohepatitis, NASH), hypertension (systolic blood pressure ≥ 140 mm Hg or diastolic ≥ 90 mm Hg), sleep apnoea, reproductive issues (obstetric/gynaecological issues, infertility, male hypogonadism and erectile dysfunction), renal impairment (eGFR < 60 mL/min/1.73m^2^), and joint pain were assessed. These measurements were recorded at referral (‘pre-surgery’) and collected from patients at 1, 3, 6, and 12-month follow-up. Further, patient-reported outcomes included health-related quality of life (HRQoL), measured using the Assessment of Quality of Life-4D (AQoL-4D) [23], eating behaviour, assessed by the Three Factor Eating Questionnaire (TFEQ) [24], and satisfaction with the BSI, measured by the Functional Assessment of Chronic Illness Therapy-Treatment Satisfaction General instrument (FACIT-TS-G) [25]. To facilitate comparisons, TFEQ scores were normalised to a 0–100 scale.

### Assessment/Validation

#### Statistical analyses

Descriptive statistics (frequencies, mean ± SD) for clinical measures and patient-reported health outcomes were computed at five time points (baseline/referral, 1, 3, 6 and 12 months) and evaluated for significance in trends. A Chi-squared test was used to access relationships between nominal or ordinal dependent variables across categories. Likewise, when examining differences over the means of interval dependant variables a one-way ANOVA approach using the GLM procedure was used. All analyses were performed using SAS (version 9.4; SAS Institute, Cary, NC, USA).

## Results

### Study population

The population for the current evaluation comprised 212 patients who consented to be assessed within the BSI (original sample), 130 of whom had complete follow-up to 12 months post-surgery (study sample). This follow-up rate was due to the COVID-19 pandemic interruption. At the time of writing, 43 patients referred to the BSI were still under review for surgery and 42 others had been excluded for patient-initiated appointment cancellations (38.1%, n = 16), smoking (30.9%; n = 13), mental health (9.5%, n = 4), and other medical reasons (21.4%, n = 9).

The 130 patients who underwent surgery and completed follow-up were 18–66 years old (mean 52.4 ± 8.1 years); 58.5% were female and 14.6% were Indigenous Australians. The cohort had an average body weight of 120.5 ± 19.3 kg and average BMI of 43.0 ± 6.7 kg/m^2^. All patients had Class I (11.5%), II (26.9%) or III (61.5%) obesity. The cohort’s average HbA1c was 8.6 ± 1.4% (7.0 ± 15.3 mmol/mol) and most patients (63%, n = 82/130) had diabetes for at least 8 years prior to referral. Patients reported multiple chronic conditions, the most prevalent of which were dyslipidaemia (81.5%, n = 106/130), sleep apnoea (68.6%, n = 70/102), and hypertension (88.5%, n = 115/130) (Table 1).

**Table 1:** Clinical characteristics and outcomes of patients who underwent bariatric surgery and had follow-up data at 12 months post-surgery (N = 130).

Patient characteristics	Pre-surgery, mean ± SD or n (%)	12 months, mean ± SD or n (%)	p-value
Age, years	52.4 ± 8.1	-	
Women	76 (58.5%)	-	
Indigenous patients	19 (14.6%)	-	
Employment status, pre-surgery			
Unemployed/Not in labour force	78 (60.0%)	-	
Part-time employment	18 (13.8%)	-	
Full-time employment	34 (26.1%)	-	
Surgery type			
Gastric bypass	89 (68.5%)	-	
Sleeve gastrectomy	41 (31.5%)	-	
Body weight, kgs	120.5 ± 19.3	92.1 ± 18.1	<0.0001^a^
Body mass index, kg/m^2^	43.0 ± 6.7	32.8 ± 6.1	<0.0001^a^
Obesity categories, n = 118			<0.0001^a^
Normal Weight (BMI 18.5–24.9)	0 (0%)	8 (6.1%)	
Overweight (BMI: 25–29.9)	0 (0%)	42 (32.3%)	
Class I Obesity (BMI: 30–34.9)	15 (11.5%)	40 (30.8%)	
Class II Obesity (BMI: 35–39.9)	35 (26.9%)	21 (16.1%)	
Class III Obesity (BMI ≥ 40)	80 (61.5%)	19 (14.6%)	
**Diabetes**			
Duration before referral			
< 4 years	14 (10.8%)	-	
4–8 years	34 (26.2%)	-	
> 8 years	82 (63.0%)	-	
Glycated haemoglobin (HbA1c), %	8.6 ± 1.4	6.5 ± 1.2	<0.0001^a^
Diabetes medications ^b^			<0.0001^a^
No medications	0 (0%)	63 (48.8%)	
Oral medications only	47 (36.1%)	53 (41.1%)	
Oral medication & insulin	79 (60.7%)	12 (9.3%)	
Insulin only	4 (3.1%)	1 (0.8%)	
Insulin dosage, units/day	120.6 ± 104.5	31.8 ± 15.4	<0.0001^a^
**Comorbidities**			
Hypertension ^c^	115 (88.5%)	70 (55.6%)	<0.0001^a^
Sleep apnoea ^d^			0.16
No	32 (31.4%)	30 (33.3%)	
Yes, no CPAP	15 (14.7%)	22 (24.4%)	
Yes, on CPAP	55 (53.9%)	38 (42.2%)	
Dyslipidaemia ^e^			0.72
No	24 (18.5%)	24 (21.6%)	
Yes, no medication	13 (10.0%)	13 (11.7%)	
Yes, on medication	93 (71.5%)	74 (66.7%)	
Joint pain ^f^	73 (57.0%)	54 (48.2%)	0.17
Non-alcoholic steatohepatitis (NASH) ^g^	17 (13.1%)	5 (4.4%)	0.02^a^
Renal impairment (eGFR < 60) ^h^	8 (6.1%)	3 (2.3%)	0.13
Reproductive issues ^i^	29 (22.3%)	22 (18.2%)	0.42

Improvement of comorbid conditions at 12 months post-surgery was only examined in those patients who reported the condition prior to surgery. CPAP–continuous positive airway pressure; eGFR–estimated glomerular filtration rate; SBP–systolic blood pressure (mm Hg); DBP–diastolic blood pressure (mm Hg).

^a^ Statistically significant, p-value < 0.05; borderline significant, p-value ≤ 0.10;

^b^ 12 months post-surgery n = 129;

^c^ 12 months post-surgery n = 126;

^d^ Pre-surgery n = 102, 12 months post-surgery n = 90;

^e^ 12 months post-surgery n = 111;

^f^ Pre-surgery n = 128, 12 months post-surgery n = 112;

^g^ 12 months post-surgery n = 114;

^h^ 12 months post-surgery n = 128;

^i^ 12 months post-surgery n = 121.

### Clinical outcomes

Table 1 presents the clinical outcomes of the study sample patients who had follow-up data at 12 months post-surgery—up to 130 (61.3%) of the 212 patients who underwent surgery between December 2017 and August 2020. Comparison of the characteristics of this 130-patient study sample to those of the full cohort of 212 patients (see S1 Table in S2 File) found no significant differences, suggesting that the outcomes observed are likely representative of the full sample. Further, comparison of patient outcomes by sex and Indigenous status (S2 Table in S2 File) found no significant difference between groups, which indicates that there is no effect of either sex or ethnicity in these results.

#### Improvement in body weight

**Fig 1:**
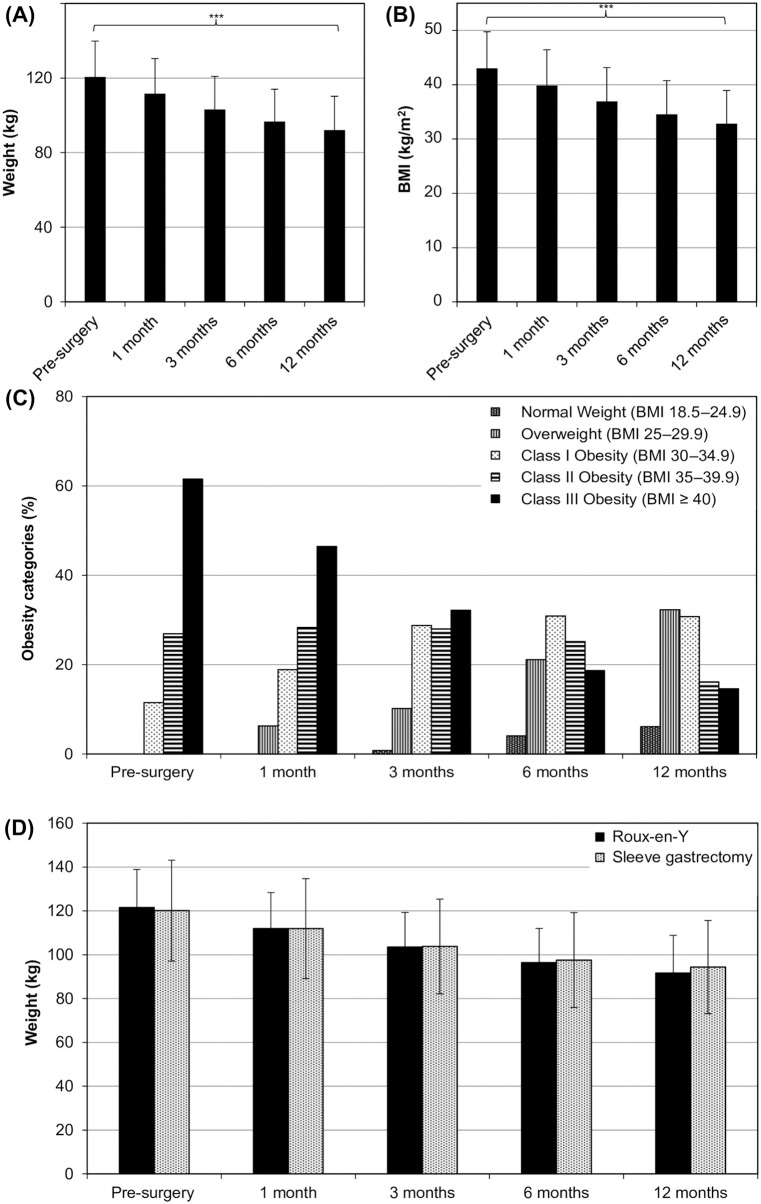
Changes to body weight and body mass index following bariatric surgery. (A) Body weight and (B) BMI were measured prior to bariatric surgery and at 1-, 3-, 6-, and 12-month follow-ups and (C) the patient distribution across obesity classes was tracked throughout this period. Similarly, (D) the weight loss of patients who underwent different surgical procedures was tracked. N = 130; 89 patients had RYGB, while 41 underwent sleeve gastrectomy. *** p < 0.001.

In the 12 months following surgery, patients’ average body weight decreased steadily (Fig 1A) from 120.5 kg to 92.1 kg, corresponding to a 23.6% reduction in average BMI and redistribution of the patients between obesity categories (Fig 1B, Table 1). Fig 1C tracks patients’ obesity status throughout the follow-up period and shows a significant decrease in the proportion of patients with class III obesity (61.5% pre-surgery v. 14.6% at 12 months) and a shift towards the overweight (0% pre-surgery v. 32.3% at 12 months) and normal weight (0% pre-surgery v. 6.2% at 12 months) categories. Although weight loss was similar in patients who underwent RYGB or SG for the first 3 months post-surgery, weight loss in RYGB patients was greater from 6 to 12 months post-surgery and ultimately resulted in 3.1% more weight lost at 12 months (24.5% for RYGB v. 21.4% for SG, p = 0.06) (Fig 1D, S3 Table in S2 File). We note that 19 patients still had class III obesity (BMI ≥ 45) at 12 months post-surgery (Fig 1 and S4 Table in S2 File). While these patients did lose weight and reduce BMI (BMI 43.8 ± 4.1 kg/m^2^ post-surgery v. 52.4 ± 5.6 kg/m^2^ pre-surgery; a 15.7 ± 10.4% reduction), the resulting decreases were insufficient to move patients between categories. In contrast, two patients progressed from having class III obesity to normal weight (S4 Table in S2 File), achieving an overall BMI change of 45.4 ± 6.0%.

#### Improvement in diabetes profiles

**Fig 2:**
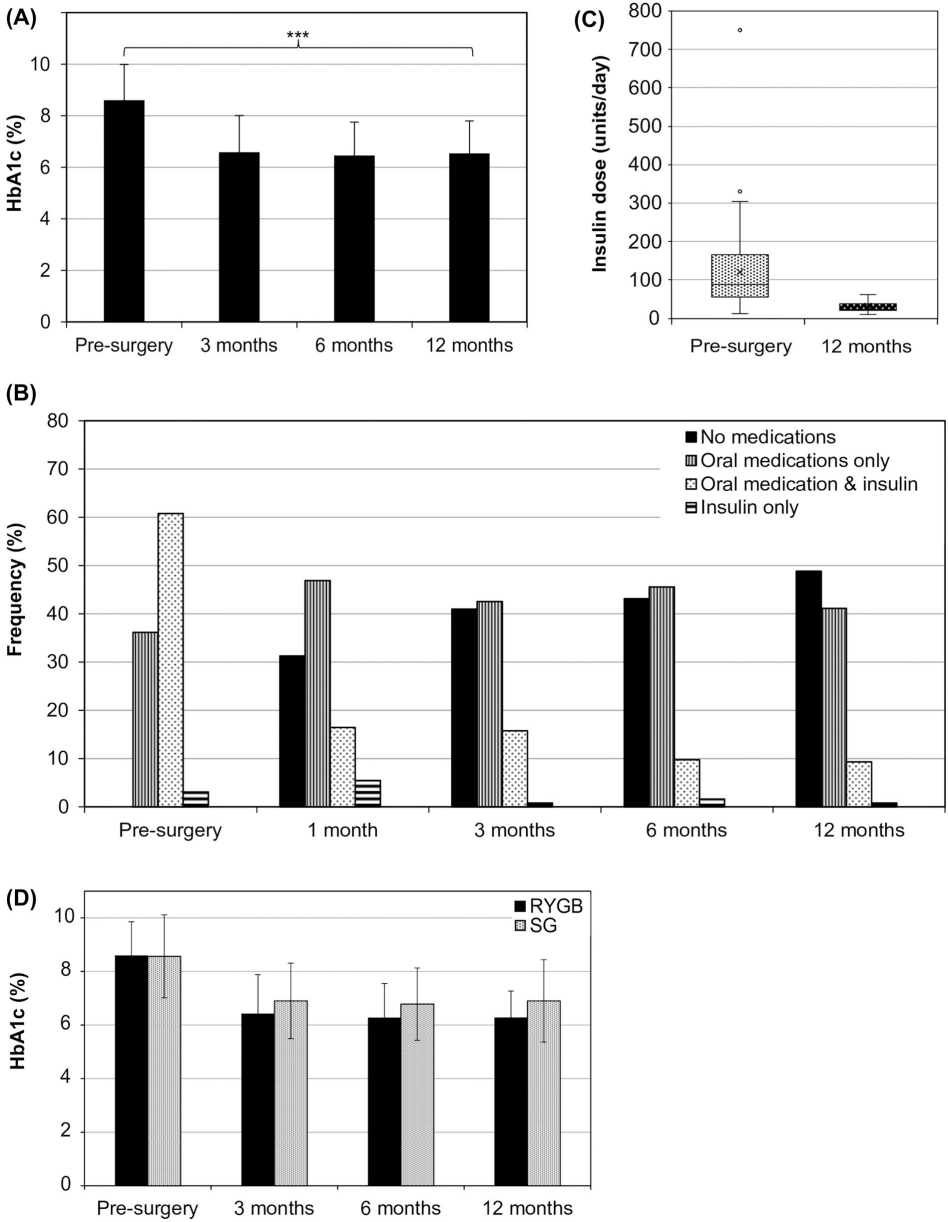
Changes in diabetes status and treatment following bariatric surgery. Changes in patients (A) glycated haemoglobin (HbA1c) were tracked before surgery and at 1-, 3-, 6-, and 12-month follow-ups (N = 130). Multiple (B) changes to patients’ diabetes medication requirements occurred post-surgery (N = 130). Patients’ (C) average insulin dosage decreased and some discontinued insulin use altogether (pre-surgery N = 82; 12 months N = 13). (D) HbA1c levels decreased at similar rates, regardless of whether patients underwent Roux-en-Y gastric bypass (RYGB) or sleeve gastrectomy (SG). *** p < 0.001.

Within the first three months following surgery, the mean HbA1c decreased by 25% from 8.6 ± 1.4% to 6.6 ± 1.5% (70 ± 15.3 mmol/mol to 48 ± 14.2 mmol/mol) (Fig 2B). This trend was maintained for the following nine months such that, within one year of surgery, 92 patients (70.2%) had HbA1c levels below 7%, with 74 patients (57%) in the healthy range of 4.1–6.5% (Diabetes Queensland 2021) (Table 1, Fig 2A). Further, RYGB patients realised significantly greater decreases in HbA1c than SG patients (24.6 ± 13.7% v. 17.8 ± 18.8%, p = 0.02; S3 Table in S2 File).

Prior to bariatric surgery, 47 patients (36.1%) were on oral medications, while the majority (79 patients, 60.7%) required both oral prescriptions and insulin to manage their diabetes (Fig 2B). Within one month of surgery, 40 patients (31.3%) had discontinued all diabetes medications and this increased to 54 (40.9%) by the end of the first year. Further, only 13 of the 83 patients who originally required insulin were still on it after 12 months and, in the remainder, the average daily dose decreased by 73.4% from 120.6 ± 104.5 units to 31.8 ± 15.4 units (Table 1, Fig 2C). While a lower proportion of the SG group was able to discontinue all medications compared to the RYGB group (35% v. 55.1% of RYGB patients), the difference was not statistically significant (p = 0.12, S3 Table in S2 File).

Patients who had diabetes for less than four years prior to referral had an average decrease in HbA1c of 37.2 ± 3.2% and all (n = 14/14) had discontinued all diabetes treatment by 12 months post-surgery (S5 Table in S2 File). In contrast, patients who had diabetes for longer than eight years decreased their HbA1c by 18.0 ± 1.7% and 31.7% (n = 26/82) were able to discontinue all diabetes medications by 12 months.

#### Improvement in other chronic disease parameters

Some patients reported improvement or resolution of comorbid conditions by 12 months post-surgery; for example, the incidence of hypertension decreased by 32.9% (88.5% pre-surgery v. 55.6% post-surgery, Table 1). While there was little change in the prevalence of sleep apnoea (68.6% pre-surgery v. 66.6% post-surgery), there was a 30.9% decrease in the number dependent on CPAP (55 pre-surgery v. 38 post-surgery). Further, NASH cases decreased by 70.5% (17/130 pre-surgery v. 5/114 post-surgery), as did 71.4% of renal impairment (7/130 pre-surgery v. 2/128 post-surgery) and 26% of joint pain cases reported pre-surgery.

#### Patient-reported outcomes

Patient-reported experiences and outcomes were collected to examine the impact of surgery on behaviour and quality of life, as well as patients’ overall satisfaction with the service. Patients reported significant changes in their eating behaviour after surgery across all three domains of the TFEQ. Higher scores indicated improvement and, within six months, emotional eating scores improved by 31.8%, uncontrolled eating was 19.8% better, and cognitive restraint had increased by 8.6% (Table 2). Particularly significant improvements (p < 0.0001) were noted in the uncontrolled and emotional eating domains, with some patients reporting perfect scores (100%) in the latter from six months post-surgery. Patients also observed an average improvement to their AQoL-4D score of 17.6% within one month of surgery (0.56 ± 0.24 pre-surgery v. 0.68 ± 0.25 at one-month follow-up, p = 0.0006), which was sustained throughout the follow-up period (Table 2). This was most likely due to significant (p = 0.002) increases in the independent living (0.84 ± 0.21 pre-surgery v. 0.93 ± 0.14 by 12 months, Table 2) and mental health (0.81 ± 0.14 pre-surgery v. 0.87 ± 0.12 at 12 months domains).

**Table 2:** Patient-reported outcomes and experiences following bariatric surgery.

Patient-reported measure	Pre-surgery	1 month	3 months	6 months	12 months	p-value
**Scaled TFEQ, 0–100, mean ± SD**						
Cognitive restraint	48.6 ± 11.2 ^a^	n.d.	n.d.	53.2 ± 13.0 ^b^	52.2 ± 13.2 ^c^	0.02^t^
Uncontrolled eating	43.0 ± 11.4 ^a^	n.d.	n.d.	53.6 ± 9.1 ^b^	54.2 ± 8.5 ^c^	<0.0001^t^
Emotional eating	41.1 ± 21.3 ^a^	n.d.	n.d.	60.3 ± 16.1 ^b^	60.9 ± 17.0 ^c^	<0.0001^t^
**AQoL-4D utility scores, mean ± SD**						
Independent living	0.85 ± 0.19 ^c^	0.85 ± 0.19 ^g^	0.91 ± 0.15 ^i^	0.92 ± 0.14 ^j^	0.93 ± 0.14 ^b^	0.002^t^
Relationships	0.83 ± 0.18 ^d^	0.89 ± 0.15 ^g^	0.89 ± 0.17 ^i^	0.88 ± 0.18 ^i^	0.88 ± 0.20 ^b^	0.09
Senses	0.81 ± 0.14 ^c^	0.94 ± 0.07^h^	0.93 ± 0.08 ^c^	0.93 ± 0.09 ^i^	0.93 ± 0.07 ^c^	0.03^t^
Mental health	0.81 ± 0.14 ^e^	0.89 ± 0.12 ^h^	0.86 ± 0.14 ^c^	0.87 ± 0.14 ^k^	0.87 ± 0.12 ^c^	0.003^t^
Total Score	0.56 ± 0.24 ^f^	0.68 ± 0.25 ^h^	0.69 ± 0.25 ^c^	0.69 ± 0.25 ^i^	0.69 ± 0.25 ^c^	0.0006^t^
**FACIT-TS-G, %**						
Effectiveness of treatment (TS1)		n.d.	69 (86.3%) ^l^	73 (90.1%) ^n^	67 (91.8%) ^p^	0.52
Side effects of treatment (TS2)		n.d.	44 (55%) ^l^	60 (74.1%) ^n^	59 (80.8%) ^p^	0.0009^t^
Doctor’s help with evaluation (TS3)		n.d.	72 (92.3%) ^m^	73 (92.4%) ^o^	70 (97.2%) ^r^	0.21
Received the right treatment (TS4)		n.d.	79 (98.8%) ^l^	79 (100%) ^o^	73 (98.6%) ^q^	1.0
Satisfied with treatment effects (TS5)		n.d.	76 (95%) ^l^	78 (96.3%) ^n^	73 (98.6%) ^q^	0.62
Recommend treatment to others (TS6)		n.d.	76 (95%) ^l^	78 (96.3%) ^n^	73 (98.6%) ^q^	0.49
Would choose treatment again (TS7)		n.d.	74 (92.5%) ^l^	70 (87.5%) ^l^	72 (98.6%) ^p^	0.03
Overall rating for treatment (TS8)		n.d.	78 (97.5%) ^l^	79 (97.5%) ^n^	73 (98.6%) ^q^	1.00

AQoL-4D and scaled TFEQ scores are mean values ± SD. FACIT-TS-G data are frequencies (%) of patients who selected the better and best options. Raw TFEQ scored were normalised to a 0–100 scale.

^a^ n = 112,

^b^ n = 95,

^c^ n = 93,

^d^ n = 94,

^e^ n = 92,

^f^ n = 91,

^g^ n = 85,

^h^ n = 82,

^i^ n = 96,

^j^ n = 98,

^k^ n = 97,

^l^ n = 80,

^m^ n = 78,

^n^ n = 81,

^o^ n = 79,

^p^ n = 73,

^q^ n = 74,

^r^ n = 72,

^t^ Statistically significant: p-value <0.05.

Patients’ satisfaction with the service and surgery was measured with the FACIT-TS-G and the frequency of patients selecting the ‘better’ or ‘best’ options was considered indicative of satisfaction. While overall approval for the treatment remained above 97% from three months post-surgery, satisfaction with the surgical side effects was initially low (55% at six months, Table 2). This indicates that 45% of patients found the side effects equal to or worse than their expectations in the months following surgery and while 80.8% of patients were satisfied with the side effects after 12 months, this area had the lowest patient satisfaction throughout follow-up. More RYGB patients experienced adverse events post-surgery than SG patients (17 v. 3, p = 0.08, S3 Table in S2 File) and FACIT-TS-G scores were further stratified by surgery type (S6 Table in S2 File) to investigate this. Patients who underwent RYGB were less satisfied with the treatment side effects than those receiving SG (52.0% v. 60.0% at three months to a maximum of 77.6% v. 87.5% at 12 months post-surgery). However, it was noted that while 3.6% fewer RYGB patients would recommend the treatment after 12 months, their overall satisfaction with bariatric surgery remained close to 100% (lowest was 98% at 3 months) throughout the follow-up period.

## Discussion

Bariatric surgery was found to have several beneficial effects on patients’ health that did not differ between sexes or ethnic groups and occurred rapidly following surgery. Weight loss began within one month. By 12 months, 37.3% of patients were no longer classified as having obesity (BMI < 30 kg/m^2^) and 67.4% had a BMI below 35 kg/m^2^. Improvement in type 2 diabetes also occurred rapidly: HbA1c levels decreased within three months and 30.8% of patients discontinued all diabetes medications within this period. By 12 months post-surgery, nearly half (48.8%) of the cohort had discontinued diabetes medications and the average insulin dose for those still requiring it had decreased by 73.6%. While the definition of diabetes remission varies between studies, most recent literature [26] requires an HbA1c of < 6.5% and cessation of all medications. We note that at the time of writing, 46 of the 130 patients (35%) with follow-up data to 12 months had both ceased all medications and achieved HbA1c levels below 6.5% for at least six months, indicating successful short-term remission of type 2 diabetes. Further, 108 patients in the present cohort were being treated at specialist diabetes management clinics prior to surgery; by 12-month follow-up, 94 (87%) had been discharged.

Consistent with previous studies [27], patients who had lived with diabetes for shorter periods achieved better diabetes outcomes. Nevertheless, we observed better glycaemic profiles in most patients following bariatric surgery, but the timing of these improvements did not coincide with weight loss. While weight loss progressed steadily throughout the follow-up period (Fig 1), patients’ HbA1c levels decreased sharply by the first follow-up at three months post-surgery and remained relatively stable thereafter. This supports previous work [28] demonstrating that remission of diabetes via bariatric surgery is not solely due to weight loss, but also the product of complex metabolic changes [29] to hormone levels, gut microbiota and signalling pathways. These factors may influence improvement in comorbid conditions, and thereby, further support diabetes remission and weight loss.

Comorbid conditions affect patients’ ability to manage their diabetes, reduce quality of life, and introduce competing financial and medication demands [30]. After bariatric surgery, comorbid conditions improved and, in some cases, resolved. This is consistent with previous studies [26] in that the greatest improvements were noted in hypertension, NASH, dyslipidaemia and join pain.

Comparisons of bariatric surgical procedures investigating which procedure offers greater benefit to patients vary in their findings: some demonstrate that those having RYGB lose more weight than SG patients and have better diabetes remission [e.g., 31, 32], while others find no significant difference between the two procedures [e.g., 33]. Although the RYGB patients in this study did lose more weight, the difference was only borderline significant (p = 0.06, S3 Table in S2 File) and no conclusions can be drawn about the greater efficacy of either surgery in weight loss within the study timeframe. In contrast, RYGB patients realised significantly (p = 0.02) greater decreases in HbA1c, suggesting an advantage for the technique in remediating diabetes, as noted by others [26]. Thus, in agreement with previous studies [31, 32, 34], these data suggest that RYGB provides better outcomes for patients with diabetes.

An important hallmark of this work is the inclusion of patient voices in the assessment process. Patient-reported outcomes and experiences are critical to assess the impact of health services and inform patient-centred care; these may not be correlated with perioperative clinical outcomes [35] and thus, provide additional insight into the nature and outcomes of care. This is particularly important for bariatric surgery, given the potential effects on mental health and the importance of patient compliance with post-surgical lifestyle changes [36]. Here, patients reported improvements to their eating behaviour, especially emotional and uncontrolled eating, which bodes well for future weight maintenance, diabetes management and HRQoL [37, 38]. As HRQoL is also linked to weight loss and resolution of comorbid conditions [39], it is perhaps unsurprising that patients reported higher HRQoL post-surgery, largely due to an improved ability to live independently.

Patients’ satisfaction with the care they receive is influenced by factors beyond their final clinical outcomes and they may value improvements differently from care providers [40]. In the current study, many patients found the surgical side-effects challenging, particularly during early follow-up, and reported lower satisfaction ratings. This appears to be linked to the surgical procedure as more RYGB patients experienced adverse events and gave lower FACIT-TS-G scores for side effects than their SG counterparts. Fortunately, their scores improved as recovery progressed and the cohort’s satisfaction with the treatment overall remained above 97.5%. Most would both choose bariatric surgery again and recommend it to others.

The paucity of long-term follow up data has been a major challenge in the use of bariatric surgery to treat obesity and type 2 diabetes—and, indeed, is a notable limitation of this study. At the time of analysis, several patients were still on the care pathway (i.e., under review for surgery or not yet at the 12-month post-surgery time point). The program experienced unavoidable interruptions to the surgical schedule as a result of the COVID-19 pandemic, which significantly affected the number of patients with complete 12-month follow-up data at the end of the study period. Funding for the study was available for a finite period and could not be extended to allow further data collection beyond the project timeframe. Thus, the low follow-up rate (130/212 patients; 61%) does not reflect poor patient retention but rather the unfortunate consequences of the pandemic. The 12-month follow-up- period itself is also significant, as some weight gain is expected around 24 months post-surgery and other studies reported fluctuations in weight and diabetes remission in the longer term. Nevertheless, these studies also observe that maintenance is consistently superior in surgical patients compared to those utilising medical/lifestyle management strategies [26].

This study utilises real-world data to provide evidence supporting the provision of bariatric surgery within the Australian public health system to treat patients with obesity, type 2 diabetes and other comorbidities. Key strengths of this work include the longitudinal study design, the consistency of results during follow-up, the use of a representative (not convenience) sample for patient-reported measures, the selection of contemporary surgical procedures, and the processes implemented to optimise patient selection. To the authors’ knowledge, it is the first of its kind in Australia because, unlike previous investigations of bariatric surgery, this study incorporated both clinical and patient-reported outcomes from its inception to ensure a comprehensive approach in which the patient’s health and wellbeing remained central. Critically, the findings suggest that the BSI delivered this treatment in a manner that met patient needs and expectations, and ultimately improved patients’ health and quality of life.

Overall, this study confirms the utility of bariatric surgery in treating morbid obesity in the Australian public health system and its positive impact on type 2 diabetes and other comorbid conditions. Consequent improvements to patients’ quality of life and eating behaviour bode well for long-term weight maintenance and diabetes remission, and, importantly, patients were ultimately satisfied with the surgery and its effects.

## Supporting information

S1 File Summary of the bariatric surgery prioritisation tool. (DOCX)

S2 File Additional clinical outcome data. (DOCX)
