# Supplementary material for: Health outcomes of patients with type 2 diabetes following bariatric surgery: Results from a publicly funded initiative
Source: PLoS One. 2023 Feb 24;18(2):e0279923. doi: 10.1371/journal.pone.0279923 (PMC9955585; doi:10.1371/journal.pone.0279923)
Supplement: S1 File — (DOCX) [file pone.0279923.s001.docx]

## S1_File. Summary of the Bariatric Surgery Prioritisation Tool

**Inclusion criteria**

Patients may be eligible if they:

- have type 2 diabetes with an HbA1c >7% despite treatment with Metformin (or alternative) plus at least one other diabetes medication; and
- have a BMI > 35 kg/m^2^; and
- are aged between 18 and 65 years; and
- are already under the care of a public hospital specialist for conditions that may be improved through bariatric surgery

**Exclusion criteria**

- Weight above 175 kg (due to logistical reasons)
- Previous bariatric surgery including lap banding
- Patients that have received or are awaiting solid organ transplants
- End stage complications of obesity (e.g., end stage cardiac disease with pulmonary hypertension, severe portal hypertension, cirrhosis)
- End stage renal failure (eGFR ≤ 15 or patient on dialysis)
- Malignancy under active treatment (excluding non-metastatic skin cancer). Previous malignancies must be 5 years disease-free.
- Any medical conditions where surgery would increase morbidity or mortality risk (e.g., portal hypertension with varices)
- Current alcohol or drug dependency (including smoking). Patient must have documented abstinence for at least 6 months prior to referral.
- Unstable mental health conditions. Patients with a history of mental illness must be stable for at least 6 months prior to referral.

**Contraindications**

- Previous gastric surgery or abdominal irradiation
- Patients on chronic immunosuppression for reasons other than organ transplant
- Other causes of obesity (e.g., neurological causes such as Prader-Willi syndrome, steroid induced)
- ASA score of 4 or above. Patients with an ASA score of 4 or above would be considered if the underlying medical conditions are mild or well-controlled.
