# Supplementary material for: Health outcomes of patients with type 2 diabetes following bariatric surgery: Results from a publicly funded initiative
Source: PLoS One. 2023 Feb 24;18(2):e0279923. doi: 10.1371/journal.pone.0279923 (PMC9955585; doi:10.1371/journal.pone.0279923)
Supplement: S2 File — (DOCX) [file pone.0279923.s002.docx]

## S2_File. Additional clinical outcome data

S1 Table. Baseline characteristics of the 212 patients who had bariatric surgery and the 130 patients in the study sample with available data at 12 months post-surgery

| **Patient characteristics** | **Original sample,  mean ± SD or n (%)**  **N = 212** | **Study sample, mean ± SD or n (%)**  **N = 130** | **p-value** |
| --- | --- | --- | --- |
| Age, years | 52.1 ± 8.6 | 52.4 ± 8.1 | 0.78 |
| Women | 125 (59.0%) | 76 (58.5%) | 0.93 |
| Indigenous patients | 41 (19.3%) | 19 (14.6%) | 0.26 |
| Employment status, pre-surgery |  |  | 0.68 |
| Unemployed/Not in labor force | 117 (55.2%) | 78 (60.0%) |  |
| Part-time employment | 32 (15.1%) | 18 (13.8%) |  |
| Full-time employment | 63 (29.7%) | 34 (26.1%) |  |
| Surgery type |  |  | 0.65 |
| Gastric bypass | 150 (70.7%) | 89 (68.5%) |  |
| Sleeve gastrectomy | 62 (29.2%) | 41 (31.5%) |  |
| Weight, kg | 120.0 ± 8.6 | 120.5 ± 19.3 | 0.81 |
| BMI, kg/m^2^ | 42.4 ± 6.5 | 43.0 ± 6.7 | 0.42 |
| Obesity categories, n = 118 |  |  | 0.76 |
| Normal Weight (BMI 18.5–24.9) | 0 (0%) | 0 (0%) |  |
| Overweight (BMI: 25–29.9) | 0 (0%) | 0 (0%) |  |
| Class I obesity (BMI: 30–34.9) | 23 (10.8%) | 15 (11.5%) |  |
| Class II obesity (BMI: 35–39.9) | 65 (30.7%) | 35 (26.9%) |  |
| Class III obesity (BMI ≥ 40) | 124 (58.5%) | 80 (61.5%) |  |
| **Diabetes** |  |  |  |
| Glycated hemoglobin (HbA1c), % | 8.7 ± 1.5 | 8.6 ± 1.4 | 0.48 |
| Diabetes medications |  |  | 0.73 |
| No medications | - | - |  |
| Oral medications only | 75 (35.4%) | 47 (36.1%) |  |
| Oral medication & insulin | 133 (62.7%) | 79 (60.8%) |  |
| Insulin only | 4 (1.9%) | 4 (3.1%) |  |
| **Comorbidities** |  |  |  |
| Hypertension | 186 (87.7%) | 115 (88.5%) |  |
| Sleep apnea |  |  | 0.97 |
| No | 51 (32.9%) | 32 (31.4%) |  |
| Yes, no CPAP | 22 (14.2%) | 15 (14.7%) |  |
| Yes, on CPAP | 82 (52.9%) | 55 (53.9%) |  |
| Dyslipidemia |  |  | 0.81 |
| No | 35 (16.5%) | 24 (18.5%) |  |
| Yes, no medication | 25 (11.8%) | 13 (10.0%) |  |
| Yes, on medication | 152 (71.7%) | 93 (71.5%) |  |
| Joint pain^b^ | 116 (55.2%) | 73 (57.0%) | 0.75 |
| Non-alcoholic steatohepatitis (NASH) | 28 (13.2%) | 17 (13.1%) | 0.97 |
| Renal impairment (eGFR < 60) | 13 (6.1%) | 8 (6.1%) | 0.99 |
| Reproductive issues | 47 (22.2%) | 29 (22.3%) |  |
| **Quality of Life**, AQoL-4D^c^ | 0.56 ± 0.26 | 0.56 ± 0.24 | 0.98 |

^a^ original sample n = 155, study sample n = 102; ^b^ original sample n = 210, study sample n = 128; ^c^ original sample n = 151, study sample n = 91.

S2 Table. Comparison of patient outcomes by sex and Indigenous status

| **Factor** | **BMI, % decrease^** | **HBA1c, % decrease^** |
| --- | --- | --- |
| Sex |  |  |
| Male, n = 54 | 22.7 ± 9.6 | 24.7 ± 14.3 |
| Female, n = 76 | 24.1 ± 8.5 | 21.0 ± 16.5 |
| p-value | 0.37 | 0.19 |
| Indigenous status |  |  |
| Indigenous | 22.0 ± 9.5 ^a^ | 26.3 ± 15.8 ^b^ |
| Non-Indigenous | 23.8 ± 8.9^c^ | 21.8 ± 15.7^d^ |
| p-value | 0.41 | 0.26 |

^ % change values were calculated as the difference from pre-surgery to 12 months post-surgery and are presented as mean ± standard deviation. Statistically significant: p < 0.05. ^a^ n = 19; ^b^ n = 18; ^c^ n = 111; ^d^ n = 110.

S3 Table. Patient outcomes and hospital information stratified by bariatric surgical procedure

| **Factor** | **Roux-en-Y**  **N = 89 (68.5%)** | **Sleeve Gastrectomy**  **N = 41 (31.5%)** | **p-value** |
| --- | --- | --- | --- |
| Age, years | 52.5 ± 7.8 | 52.0 ± 8.9 | 0.76 |
| Female | 49 (55.1%) | 27 (65.8%) | 0.25 |
| Obesity class (at the time of surgery) |  |  | 0.66 |
| Obese I | 10 (11.2%) | 5 (12.2%) |  |
| Obese II | 22 (24.7%) | 13 (31.7%) |  |
| Obese III | 57 (64.1%) | 23 (56.1%) |  |
| Adverse events | 17 (19.1%) | 3 (7.3%) | 0.08** |
| Nausea vomiting | 5 (5.6%) | 0 (0.0%) |  |
| Unplanned readmissions to hospital | 8 (9.0%) | 2 (4.9%) |  |
| Unplanned readmission to theatre | 1 (1.1%) | 1 (2.4%) |  |
| Unplanned admission to ICU | 1 (1.1%) | 0 (0.0%) |  |
| Surgical complications | 3 (3.4%) | 0 (0.0%) |  |
| Body weight |  |  |  |
| Pre-surgery | 121.1 ± 17.8 | 119.3 ± 22.4 | 0.63 |
| Post-surgery; 12 months | 91.2 ± 16.9 | 93.9 ± 20.6 |  |
| p-value |  |  |  |
| Weight loss % | 24.5 ± 9.4 | 21.4 ± 7.5 | 0.06 |
| Body mass index |  |  |  |
| Pre-surgery | 42.7 ± 6.3 | 43.6 ± 7.7 | 0.51 |
| Post-surgery; 12 months | 32.2 ± 5.7 | 34.2 ± 6.8 |  |
| p-value |  |  |  |
| BMI loss % | 24.5 ± 9.4 | 21.4 ± 7.5 | 0.06 |
| HbA1c |  |  |  |
| Pre-surgery | 8.7 ± 1.3 | 8.5 ± 1.5 | 0.49 |
| Post-surgery; 12 months | 6.4 ± 1.1 | 6.8 ± 1.5 |  |
| p-value |  |  |  |
| HbA1c change % | 24.6 ± 13.7 | 17.8 ± 18.8 | 0.02* |
| Diabetes medications: pre-surgery |  |  | 0.87 |
| No medications | 0 (0.0%) | 0 (0.0%) |  |
| Oral medications | 31 (34.8%) | 16 (39.0%) |  |
| Oral medications & insulin | 55 (61.8%) | 24 (58.5%) |  |
| Insulin alone | 3 (3.4%) | 1 (2.4%) |  |
| Diabetes medications: 12 months |  |  | 0.12 |
| No medications | 49 (55.1%) | 14 (35.0%) |  |
| Oral medications | 32 (36.0%) | 21 (52.5%) |  |
| Oral medications & insulin | 7 (7.9%) | 5 (12.5%) |  |
| Insulin alone | 1 (1.1%) | 0 (0.0%) |  |

*Statistically significant: p-value < 0.05; ** borderline significant: p-value < 0.01.

S4 Table. Change in patient BMI and HbA1c over 12 months post-surgery stratified by pre-surgery obesity category

| **Obesity category** | | **BMI** | | | | **HbA1c** | | | |
| --- | --- | --- | --- | --- | --- | --- | --- | --- | --- |
|  |  | **N = 130** | **BMI, Mean ± SD, kg/m2** | | **BMI, % decrease** | **N = 128** | **HbA1c, %, Mean ± SD** | | **HbA1c, % decrease** |
| **Pre-surgery** | **12 months** | **n** | **Pre-surgery** | **12 months** | **Mean ± SD, %** | **n** | **Pre-surgery** | **12 months** | **Mean ± SD, %** |
| Class I obesity |  | 15 | 33.6 ± 1.2 | 25.9 ± 2.4 | 23.1 ± 5.3 | 14 | 8.7 ± 1.2 | 6.6 ± 1 | 20.6 ± 15 |
|  | Normal weight | 5 | 32.6 ± 1.4 | 23.4 ± 1.5 | 28.1 ± 2.5 | 5 | 8.7 ± 0.3 | 6.4 ± 0.7 | 26.1 ± 7.6 |
|  | Overweight | 9 | 34 ± 0.7 | 26.7 ± 1.4 | 21.5 ± 3.7 | 8 | 8.8 ± 1.6 | 6.9 ± 1.2 | 16 ± 18.1 |
|  | Class I obesity | 1 | 34.9 ± 0 | 30.5 ± 0 | 12.4 ± 0 | 1 | 8.1 ± 0 | 5.7 ± 0 | 29.6 ± 0 |
| Class II obesity |  | 35 | 37.8 ± 1.3 | 29.5 ± 2.9 | 21.9 ± 7.8 | 34 | 8.8 ± 1.2 | 6.8 ± 1.5 | 22.3 ± 14 |
|  | Normal weight | 1 | 38.3 ± 0 | 23.6 ± 0 | 38.4 ± 0 | 1 | 10 ± 0 | 7.1 ± 0 | 29 ± 0 |
|  | Overweight | 21 | 37.5 ± 1.4 | 27.9 ± 1.5 | 25.6 ± 4.8 | 21 | 9.1 ± 1.2 | 7.2 ± 1.8 | 21.1 ± 16.8 |
|  | Class I obesity | 12 | 38.1 ± 1.2 | 32.1 ± 1.3 | 15.6 ± 5 | 11 | 8.3 ± 1 | 6.2 ± 0.6 | 23.7 ± 8.6 |
|  | Class II obesity | 1 | 37.7 ± 0 | 36.1 ± 0 | 4.4 ± 0 | 1 | 8.8 ± 0 | 6.6 ± 0 | 25 ± 0 |
| Class III obesity |  | 80 | 47.1 ± 5.3 | 35.6 ± 5.9 | 24.3 ± 9.9 | 80 | 8.5 ± 1.5 | 6.4 ± 1.2 | 22.9 ± 16.6 |
|  | Normal weight | 2 | 43.2 ± 3.8 | 23.5 ± 0.5 | 45.4 ± 6 | 2 | 7.4 ± 0 | 5.7 ± 1.3 | 23 ± 17.2 |
|  | Overweight | 12 | 43.8 ± 2.2 | 28.7 ± 1.3 | 34.4 ± 4.4 | 12 | 9 ± 1.6 | 5.9 ± 0.8 | 33.7 ± 11.1 |
|  | Class I obesity | 27 | 44.2 ± 2.9 | 32.9 ± 1.3 | 25.4 ± 5.6 | 27 | 8.7 ± 1.7 | 6.3 ± 0.9 | 26.3 ± 15.8 |
|  | Class II obesity | 20 | 48.1 ± 4.7 | 36.8 ± 1.2 | 22.8 ± 7.7 | 20 | 8.6 ± 1.6 | 6.7 ± 1.5 | 20.3 ± 18.7 |
|  | Class III obesity | 19 | 52.4 ± 5.6 | 43.8 ± 4.1 | 15.7 ± 10.4 | 19 | 7.7 ± 0.7 | 6.6 ± 1.2 | 14 ± 14.3 |

Obesity categories: Normal weight (BMI 18.5–24.9), Overweight (BMI 25–29.9), Class I obesity (BMI 30–34.9), Class II obesity (BMI 35–39.9), Class III obesity (BMI ≥ 40).

S5 Table. Changes to patients’ weight and diabetes from pre-surgery to 12 months post-surgery, stratified by the duration of their diabetes prior to referral

| **Parameter** | **< 4 years** | **4–8 years** | **> 8 years** | **p-value** |
| --- | --- | --- | --- | --- |
| N | 14 | 34 | 82 |  |
| **Body Weight/BMI** |  |  |  |  |
| BMI, kg/m^2^, pre-surgery | 51.9 ± 1.4 | 48.8 ± 1.3 | 44.7 ± 0.8 | < 0.001* |
| BMI, kg/m^2^, 12 months | 35.1 ± 1.0 | 33.8 ± 1.1 | 32.0 ± 0.7 | 0.03* |
| Decrease in weight/BMI, % | 25.8 ± 1.9 | 24.5 ± 1.7 | 22.7 ± 1.0 | 0.47 |
| **Diabetes** |  |  |  |  |
| HbA1c, %, pre-surgery | 8.9 ± 0.51 | 8.3 ± 0.25 | 8.6 ± 0.13 | 0.36 |
| HbA1c, %, 12 months | 5.37 ± 0.08 | 6.00 ± 0.14 | 7.00 ± 0.14 | < 0.001* |
| Decrease in HbA1c, % | 37.2 ± 3.2 | 27.0 ± 2.4 | 18.0 ± 1.7 ^a^ | < 0.001* |
| On oral medication at referral | 14 (100%) | 33 (97.1%) | 79 (96.3%) | 0.76 |
| Discontinued oral medications ^ | 14 (100%) | 23 (71.9%) | 26 (32.9%) | < 0.001* |
| On insulin at referral | 5 (35.7%) | 16 (47.1%) | 62 (75.6%) | 0.001* |
| Discontinued insulin ^ | 5 (100%) | 16 (100%) | 49 (79.0%) | 0.07 |
| No medications at 12 months ^ | 14 (100%) | 23 (69.7%) | 26 (31.7%) | < 0.001* |

Data are presented as mean ± standard deviation or N (%). ^ The percentage of patients discontinuing medications is calculated relative to the number who reported taking those medications at referral. ^a^ N = 80. *Statistically significant: p-value < 0.05.

## Additional patient-reported data

Patient satisfaction was measured by the FACIT-TS-G instrument. The percentage of patients who selected the ‘better’ or ‘best’ options, indicating satisfaction, is presented in S5 Table, stratified by surgical procedure.

S6 Table. Proportion^ of patients satisfied with treatment aspects, stratified by surgery type

| **FACIT-TS-G component** | **3 months** | | **6 months** | | **12 months** | |
| --- | --- | --- | --- | --- | --- | --- |
|  | **RYGB ^a^** | **SG^b^** | **RYGB^c^** | **SG^d^** | **RYGB^e^** | **SG^f^** |
| TS1. Effectiveness of treatment | 84.0% | 90.0% | 92.5% | 85.7% | 89.8% | 95.8% |
| TS2. Side effects of treatment | 52.0% | 60.0% | 78.8% | 70.4% | 77.6% | 87.5% |
| TS3. Doctor's help with evaluation | 89.8% | 96.6% | 86.8% | 96.4% | 97.9% | 95.8% |
| TS4. Received the right treatment | 100.0% | 96.7% | 98.1% | 96.4% | 100.0% | 96.0% |
| TS5. Satisfied with treatment effects | 100.0% | 96.7% | 96.2% | 96.4% | 100.0% | 96.0% |
| TS6. Recommend treatment to others | 96.0% | 93.3% | 96.2% | 96.4% | 98.0% | 100.0% |
| TS7. Would choose treatment again | 90.0% | 96.7% | 86.5% | 89.3% | 98.0% | 100.0% |
| TS8. Overall rating for treatment | 98.0% | 96.7% | 98.1% | 96.4% | 100.0% | 96.0% |

^% of patients who selected options equivalent to ‘better’ or ‘best’; ^a^ N = 50, ^b^ N = 30, ^c^ N = 53, ^d^ N = 28, ^e^ N = 49, ^f^ N = 25.
